# The Antibiotic Bacitracin Protects Human Intestinal Epithelial Cells and Stem Cell-Derived Intestinal Organoids from *Clostridium difficile* Toxin TcdB

**DOI:** 10.1155/2019/4149762

**Published:** 2019-08-05

**Authors:** Ziyu Zhu, Leonie Schnell, Bastian Müller, Martin Müller, Panagiotis Papatheodorou, Holger Barth

**Affiliations:** ^1^Institute of Pharmacology and Toxicology, University of Ulm Medical Center, 89081 Ulm, Germany; ^2^Department of Internal Medicine I, University of Ulm Medical Center, 89081 Ulm, Germany

## Abstract

Bacitracin is an established antibiotic for local application and inhibits the cell wall synthesis of Gram-positive bacteria. Recently, we discovered a completely different mode of action of bacitracin and reported that this drug protects human cells from intoxication by a variety of medically relevant bacterial protein toxins including CDT, the binary actin ADP-ribosylating toxin of *Clostridium* (*C.*) *difficile*. Bacitracin prevents the transport of CDT into the cytosol of target cells, most likely by inhibiting the transport function of the binding subunit of this toxin. Here, we tested the effect of bacitracin towards TcdB, a major virulence factor of *C. difficile* contributing to severe *C. difficile*-associated diseases (CDAD) including pseudomembranous colitis. Bacitracin protected stem cell-derived human intestinal organoids as well as human gut epithelial cells from intoxication with TcdB. Moreover, it prevented the TcdB-induced disruption of epithelia formed by gut epithelium cells *in vitro* and maintained the barrier function as detected by measuring transepithelial electrical resistance (TEER). In the presence of bacitracin, TcdB was not able reach its substrate Rac1 in the cytosol of human epithelial cells, most likely because its pH-dependent transport across cell membranes into the cytosol is decreased by bacitracin. In conclusion, in addition to its direct antibiotic activity against *C. difficile* and its inhibitory effect towards the toxin CDT, bacitracin neutralizes the exotoxin TcdB of this important pathogenic bacterium.

## 1. Introduction

Stem cell-based 3D organoid culture techniques have previously been used to establish new approaches for disease modeling by utilizing complex human organ-like structures that exhibit specific features of terminal differentiated tissue types. In contrast to monolayer cell culture lines, stem cell-derived organoids include all relevant differentiated cell types of a particular organ [1]. The usage of iPSC-derived organoids for dissecting both the toxic mechanisms and potential pharmacological inhibitors of bacterial protein toxins represents a promising preclinical *in vitro* approach to screen for new therapeutic toxin inhibitors [2, 3].

*Clostridium difficile* infections (CDI) are of major medical interest in Western countries because they cause up to 25% nosocomial cases of antibiotic-associated diarrhea [4–8]. The clinical symptoms of *C. difficile*-associated diseases (CDAD) range from mild diarrhea to life-threatening pseudomembranous colitis. *C. difficile* is a Gram-positive, anaerobic spore-forming bacterium that produces protein toxins, which represent highly potent virulence factors and are directly attributed to CDAD [9–12]. The toxins A (TcdA) and B (TcdB) are single-chain toxins that enter human intestinal cells via receptor-mediated endocytosis mediated by their binding/transport subunits [13] and finally deliver their enzymatically active subunits from acidified endosomal vesicles into the cytosol of their target cells [14, 15] (for review see [16]). There, the enzyme subunits monoglucosylate Rho- and Ras-GTPases [17], which inhibits signal transduction via these central molecular switches (for review see [16]). The TcdB-mediated inhibition of Rho-GTPases results in the destruction of the actin cytoskeleton with several cytopathic effects including colonocyte death and disruption of the intestinal barrier function [13]. Some hypervirulent *C. difficile* strains produce in addition to TcdA and TcdB a third toxin, the binary CDT (*Clostridium difficile transferase*) [18, 19]. The enzyme subunit of CDT mono-ADP-ribosylates G-actin in cells, which results in the destruction of the cytoskeleton accompanied by alterations in microtubule structures (for review see [16]). Because the enzyme activity of the *C. difficile* toxins in the cytosol of human intestinal epithelial cells is the prerequisite for the cytotoxic effects and the clinical symptoms associated with CDAD, specific pharmacological inhibitors that protect human gut cells from the toxic effects of the toxins are needed for the development of novel therapeutic options.

Bacitracin (Bac) is an established antibacterial drug that inhibits the cell wall synthesis of Gram-positive bacteria after topic application as it is not efficiently resorbed from the gut [20–23]. Bac was already clinically tested for efficacy in patients with *C. difficile*-induced diarrhea in comparison with vancomycin. Interestingly, Bac is equivalent in resolving clinical symptoms but inferior in eradicating *C. difficile*. Potential toxin-neutralizing effects of Bac combined with inferior antibiotic potency for *C. difficile* might be an explanation for this observation [24].

Recently, we reported that Bac in addition to its antibacterial activity neutralizes a variety of medically relevant bacterial protein toxins and protects cultured human cells from intoxication by these toxins including binary toxins such as anthrax lethal toxin and the actin ADP-ribosylating toxin CDT of *C. difficile* [25]. We discovered that Bac prevents the pH-mediated transport of the enzyme subunits of these toxins across endosomal membranes into the cytosol of target cells, most likely by inhibiting the essential membrane transport function of their binding/transport subunits [25].

Here, we show that Bac protects human intestinal epithelial cells as well as stem cell-derived human intestinal organoids from intoxication with TcdB. Moreover, Bac prevents the TcdB-induced disruption of epithelia formed by intestinal epithelial cells *in vitro* and maintains the barrier function in the presence of TcdB as detected by measuring transepithelial electrical resistance (TEER). By investigating the underlying molecular mechanism in more detail, we found that Bac has no effect on the enzyme activity of TcdB but decreases the pH-dependent transport of the toxin into the cytosol of target cells and thereby the TcdB-catalyzed glucosylation of its substrate Rac1 in the cytosol of human epithelial cells.

## 2. Materials and Methods

### 2.1. Materials

MEM and Dulbecco's modified Eagle medium (DMEM) cell culture medium and fetal calf serum (FCS) were from Invitrogen (Karlsruhe, Germany) and materials for cell culture from TPP (Trasadingen, Switzerland). Complete® protease inhibitor was supplied by Roche (Mannheim, Germany), protein weight marker PageRuler Prestained Protein Ladder® by Thermo Fisher Scientific Inc. (Waltham, MA, USA). Baf A1 and Bac were obtained from Calbiochem (Bad Soden, Germany), and the enhanced chemiluminescence (ECL) system was from Millipore (Schwalbach, Germany). The antibody detecting nonglucosylated Rac1 [26] was from BD Biosciences (Franklin Lakes, NJ, USA), against G-actin from Thermo Fisher Scientific Inc. (Waltham, MA, USA), and the secondary peroxidase-coupled anti-mouse antibody was from Santa Cruz Biotechnology (Heidelberg, Germany). TcdB was purified as described [13].

### 2.2. Cell Culture and Cytotoxicity Assays

HeLa cells from DSMZ (Braunschweig, Germany) were cultivated in MEM containing 10% heat-inactivated FCS, 1.5 g/l sodium bicarbonate, 1 mM sodium pyruvate, 2 mM L-glutamine, 0.1 mM nonessential amino acids, and 1% penicillin-streptomycin at 37°C and 5% CO_2_. CaCo-2 (human epithelial colorectal adenocarcinoma cells ATCC®HTB-37™) cells were cultivated in DMEM including 10% heat-inactivated FCS, 1 mM sodium pyruvate, 0.1 mM nonessential amino acids, and 1% penicillin-streptomycin at 37°C and 5% CO_2_. Cells were trypsinized and reseeded for at most 30 times. For cytotoxicity experiments, CaCo-2 cells were seeded in culture dishes and incubated in complete medium. For the analysis of its inhibitory activity, cells were preincubated with Bac for 30 min followed by the addition of TcdB and further incubation at 37°C with the toxin in the presence or absence of Bac. According to the indicated incubation times, cell rounding mediated by the activity of the respective toxin in the host cell cytosol as specific endpoint of the intoxication process was visualized by using a Zeiss Axiovert 40 CFL inverted microscope (Oberkochen, Germany) and a 20x objective with a Jenoptik progress C10 CCD camera (Jena, Germany). The percentages of round, i.e., intoxicated, cells were calculated from the pictures.

### 2.3. SDS-PAGE and Immunoblot Analysis

For immunoblot analysis, samples were denatured at 95°C in reducing sample buffer and subjected to SDS-PAGE. Thereafter, the proteins were transferred to a nitrocellulose membrane (Whatman, Dassel, Germany), and then, blocking of the membrane was performed for 1 h with 5% nonfat dry milk in phosphate-buffered saline (PBS) containing 0.1% Tween-20 (PBST). Nonglucosylated Rac1 was detected by using a specific antibody for nonglucosylated Rac1 [26] and visualized by using the ECL system according to the manufacturer's instructions.

### 2.4. *In Vitro* Glucosylation of Rac1 from CaCo-2 Lysate by TcdA and TcdB

TcdB (60 ng/*μ*l = 220 nM) was incubated with Bac (3 mM) for 30 min at 37°C. Then, whole CaCo-2 lysate (40 *μ*g of protein) was added and samples (25 *μ*l final volume) were incubated for 120 min at the same conditions. Following this, the samples were subjected to SDS-PAGE and blotted onto a nitrocellulose membrane, and the nonglucosylated Rac1 was detected with specific antibodies and the ECL system. Detection of G-actin was performed in addition to confirm comparable protein loading and blotting. Therefore, after blotting of the samples onto the nitrocellulose membrane, incubation with anti-actin followed by incubation with goat anti-rabbit IgG HRP was performed prior to the detection by the ECL system.

### 2.5. Analysis of the Glucosylation Status of Rac1 in TcdB-Treated Cells

CaCo-2 cells were challenged with TcdB after preincubation with a dose-titration of Bac (3, 1, and 0.3 mM) for 30 min at 37°C. For control, cells were left untreated or treated only with Bac (3 mM). After further incubation at 37°C, cells were washed with PBS and lysed by freeze thawing, and cell lysates were then transferred to SDS-PAGE. Subsequently, the nonglucosylated Rac1 from these cells was detected by Western blotting. Comparable amount of protein loading was confirmed by the detection of G-actin.

### 2.6. Toxin Translocation Assay across the Cytoplasmic Membrane

The pH-dependent toxin translocation of TcdB across the cytoplasmic membranes of intact HeLa cells was performed as described earlier [14] with some modifications of the experimental setup. In brief, HeLa cells were preincubated for 30 min at 37°C in serum-free medium containing 200 nM bafilomycin (Baf) A1, an inhibitor of the v-ATPase that inhibits acidification of the endosomal lumen in cells and thereby the pH-dependent transport of the enzyme domain of TcdB from endosomes into the cytosol [14]. Following this, for binding of the respective toxin to the cell surface, cells were incubated at 4°C with TcdB (200 ng/ml). Subsequently, cells were exposed to an acidic pulse (pH 3.8) at 37°C to trigger pH-driven membrane insertion and translocation of TcdB, respectively, across the membranes into the host cell cytosol. During this step, the acidic medium either contained Bac (1 mM) or not, in order to test the effect of Bac on the pH-driven membrane transport of TcdB. Thereafter, all cells were further incubated at 37°C in neutral medium containing FCS and 200 nM Baf A1. The toxin-induced cell rounding as specific and sensitive endpoint for translocation into the cytosol was monitored by microscopy and quantified by calculating the percentage of round cells from microscope images.

### 2.7. Transepithelial Electrical Resistance Measurements

Transepithelial electrical resistance (TEER) measurements were performed by impedance spectrometry using the CellZScope (NanoAnalytics GmbH, Muenster, Germany) as described [25]. In brief, 3 × 10^4^ CaCo-2 cells were seeded on 24-well cell culture inserts (Transwell® Permeable Supports, PET, 0.4 *μ*m from Corning GmbH, Wiesbaden, Germany) and incubated at 37°C and 5% CO_2_ until a confluent monolayer was obtained. Cells were preincubated with Bac (2 mM) for 30 min before the addition of TcdB into the upper compartment. Measurements were conducted every 35 min over a period of 20 h. Data acquisition and analysis were performed using the software provided with the CellZScope (NanoAnalytics, Münster, Germany).

### 2.8. Analysis of the Glucosylation Status of Rac1 in Human Intestinal Organoids

The use of human material in this study has been approved by the ethical committee of the Ulm University (No. 0148/2009) and Tübingen University (638/2013BO1) and is in compliance with the guidelines of the Federal Government of Germany and the Declaration of Helsinki concerning Ethical Principles for Medical Research Involving Human Subjects. A healthy volunteer gave a written informed consent. iPSC-derived intestinal organoids were generated according to an established protocol [27]. Human intestinal organoids derived from stem cells from plugged human hair were incubated in Matrigel and medium [27, 28]. Intoxication experiments with organoids were performed as described [2]. In brief, organoids were placed in a 24-well plate. Prior to intoxication, organoids were preincubated with Bac (1 mM) or left untreated for control for 30 min at 37°C. Then, TcdB (60 ng/ml) was added for 3 h at 37°C. After washing, organoids were released from Matrigel, washed again, and then fixed by 4% PFA in 10% sucrose solution for 20 min at RT. After washing and incubation in 25% sucrose solution overnight at 4°C, organoids were placed in cryomolds, embedded in tissue-tek OCT compound, and frozen in liquid nitrogen. Frozen sections (8 *μ*m) were made and dried overnight at RT. After rehydration, samples were blocked (10% goat serum), permeabilized (0.2% Triton X100 in PBS), and treated with an antibody against nonglucosylated Rac1 overnight at 4°C. Fluorescence-labeled secondary antibody in 5% goat serum and 0.1% Triton X100 in PBS was added for 1 h at RT. Moreover, nuclei were stained with Hoechst® 33342 and F-actin with phalloidin-FITC. Fluorescence images were taken with the iMic Digital Microscope from FEI.

### 2.9. Reproducibility of Experiments and Statistics

All experiments were performed independently at least two times, and results from representative experiments are shown in the figures. Values (in most cases *n* = 3) are calculated as mean ± standard deviation (SD) or standard error of the mean (SEM) as indicated in the individual experiment using the Prism4 Software (GraphPad Software, La Jolla, USA). Where cropped Western blots are displayed, the blot panels were cut and recombined for presentation purposes only. All corresponding protein bands were originally detected on the same membrane and X-ray film.

## 3. Results and Discussion

### 3.1. Bac Protects Human Intestinal Epithelial Cells from Intoxication with TcdB

**Figure 1:**
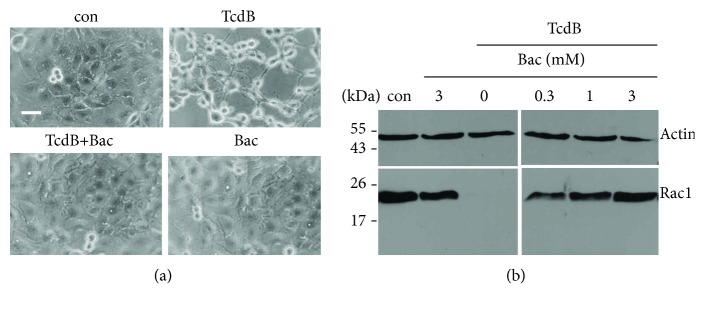
Bac protects human intestinal epithelial cells from intoxication with TcdB. (a) CaCo-2 cells were preincubated for 30 min in serum-containing medium with or without Bac (1 mM). TcdB (6 ng/ml) was added into the medium and cells were further incubated. For control (con), cells were left untreated. Representative pictures of the cells after 5 h of incubation are shown. The scale bar represents 50 *μ*m. (b) Bac prevents Rac1 glucosylation by TcdB in living cells. CaCo-2 cells were preincubated at 37°C with 0, 0.3, 1, or 3 mM Bac and TcdA (6 ng/ml) was added. For control, cells were left untreated (con) or treated with 3 mM Bac in the absence of TcdB. After 5 h of incubation, cells were washed and lysed and the amount of nonglucosylated Rac1 was analyzed by Western blotting. Actin was detected in the same blot to confirm comparable protein loading.

Prompted by our recent observation that Bac neutralizes a variety of bacterial protein toxins including CDT, we analyzed whether Bac has an effect on the intoxication of human intestinal epithelial cells with TcdB. Therefore, CaCo-2 cells were challenged with TcdB in the presence and absence of Bac and the TcdB-mediated cell rounding was analyzed as well-established specific and highly sensitive endpoint to monitor the activity of TcdB in the cytosol of these cells. As shown in Figure 1(a), all cells were round after TcdB treatment while Bac protected the cells from the cytotoxic effects. Bac alone had no effect on cell morphology.

This finding was confirmed by analyzing the glucosylation status of Rac1 from cells treated with TcdB in the absence or presence of Bac. The result shown in Figure 1(b) demonstrates that unmodified Rac1 was detected when cells were treated with TcdB in the presence of increasing concentrations of Bac, while Rac1 was completely modified in the cells when they were treated with TcdB in the absence of Bac. Bac alone had no effect on the glucosylation status of Rac1. This result clearly indicates that TcdB was not able to modify Rac1 in the cytosol of cells when Bac was present.

However, the results gave no hint whether this was the case because Bac inhibited the TcdB-catalyzed glucosylation of Rac1 in the cytosol of the cells or the uptake of the catalytic subunit of TcdB into the cytosol. To address this question, the effect of Bac on the TcdB-catalyzed glucosylation of Rac1 was analyzed *in vitro*. To this end, TcdB was preincubated with or without Bac and then CaCo-2 lysate which served as a source for Rac1 and all essential cofactors was added. Following incubation, the nonglucosylated Rac1 was detected by Western blotting with a specific antibody that selectively recognizes the unmodified but not the glucosylated Rac1 [26]. Bac did not decrease the TcdB-catalyzed glucosylation of Rac1, strongly suggesting that Bac prevents the uptake of the catalytic subunit of TcdB into the cytosol, i.e., Bac might interfere with the cellular uptake of TcdB.

**Figure 2:**
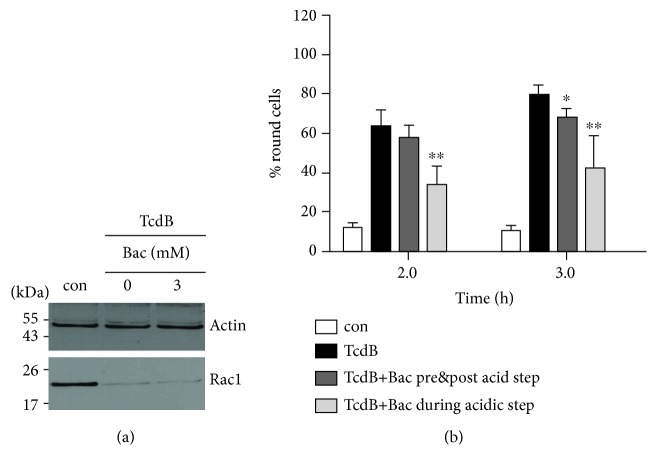
Bac does not inhibit the TcdB-catalyzed glucosylation of Rac1 but decreases the pH-dependent membrane translocation of TcdB in human epithelial cells. (a) Bac has no effect on the TcdB-catalyzed glucosylation of Rac1 *in vitro*. TcdB (300 ng) was preincubated in the presence or absence of Bac (3 mM) for 30 min at 37°C. Then, CaCo-2 lysate (40 *μ*g protein) was added and the samples were further incubated for 2 h at 37°C. Nonglucosylated Rac1 was detected by Western blotting. Actin was detected to confirm comparable protein loading. (b) Bac decreases the pH-dependent translocation of TcdB across cell membranes into the cytosol. HeLa were preincubated in serum-free medium containing Baf A1 (200 nM) for 30 min at 37°C. Afterwards, cells were kept for 40 min on ice prior to application of TcdB (200 ng/ml) and further incubation for 40 min at 4°C to enable TcdB binding to its cell surface receptor but to prevent the “normal” uptake of the cell-bound TcdB via acidified endosomal vesicles. Subsequently, cells were exposed for 5 min at 37°C to an acidic pulse (pH 3.8) to trigger the pH-dependent TcdB translocation into the cytosol. For control (con), additional toxin-treated cells were incubated at neutral conditions (pH 7.5). Following the acidic pulse, all samples were further incubated at 37°C in pH-neutral medium containing serum and Baf A1 (200 nM). Pictures from the cells were taken and the percentages of round cells calculated from the pictures. Values are given as mean ± SD (*n* = 3). Statistical significance between toxins' groups and the bacitracin effect groups was tested using Student's *t*-test (^∗^*p* < 0.05, ^∗∗^*p* < 0.01). To analyze the effect of Bac (1 mM) on the binding of TcdB to the cells, Bac was applied to the cells either 30 min prior and during TcdB binding and after the acidic step. To test the effect of Bac on the pH-dependent translocation of TcdB across the cytoplasmic membrane, Bac (1 mM) was only applied during the acidic step.

To test this hypothesis, we analyzed the cellular uptake of TcdB in the presence and absence of Bac by performing a well-established assay that mimics the “normal” transport of TcdB across endosomal membranes on the surface of adherent epithelial cells. In brief, the binding of TcdB to its receptor was enabled by incubating the cells with TcdB at 4°C. At 4°C, binding but not internalization of the toxin is possible. Subsequently, the cells were washed to remove unbound toxin and exposed for 5 min at 37°C to a warm and acidic medium (pH 3.8). This acidic pulse triggers the pH-dependent translocation of the cell-bound TcdB across the cytoplasmic membrane and thereby the transport of its catalytic subunit B into the cytosol, which results in toxin-induced cell rounding after further incubation of the cells in neutral and warm medium (Figure 2(b), black bars), due to the inhibition of Rho-GTPases in the cytosol by the catalytic subunit of TcdB. In contrast, TcdB-treated cells which were not exposed to the acidic step did not round up (Figure 2(b), white bars), demonstrating that the acidic pulse is essential to trigger the translocation of cell-bound toxin into the cytosol. Thus, this highly specific and sensitive endpoint clearly indicates whether the catalytic subunit of TcdB reaches the cytosol. To prevent the “normal” uptake of the cell-bound TcdB via acidified endosomal vesicles, bafilomycin A1 which inhibits endosomal acidification was permanently present in the media.

Most of the cells, which were treated that way in the absence of Bac, were round after 2 h of incubation after the acidic pulse (Figure 2(b), black bars). This was comparable for cells which were treated with Bac only prior and after, but not during the acidic pulse (Figure 2(b), dark grey bars), indicating that Bac did not relevantly reduce the binding of TcdB to cells. However, when cells were treated with Bac only during the acidic pulse, there was a significantly reduced amount of round cells after 2 and 3 h, indicating that less TcdB enzyme activity reached the cytosol of cells under these conditions. Since Bac had no inhibitory effect on the enzyme activity of TcdB (Figure 2(a)), this result strongly suggests that the presence of Bac during the acidic pulse decreases the amount of the catalytic subunit of TcdB in the cytosol by inhibiting the pH-dependent membrane transport step of TcdB.

In a recent study, we already excluded that Bac inhibits endocytic mechanisms of cells [25]. In conclusion, Bac inhibits the pH-dependent membrane translocation of the catalytic subunit of TcdB, which is in accordance with the recent findings for other bacterial toxins that also deliver their catalytic subunit from acidic endosomes into the cytosol.

### 3.2. Bac Protects CaCo-2 Monolayers from TcdB and Preserves Their Epithelial Integrity

**Figure 3:**
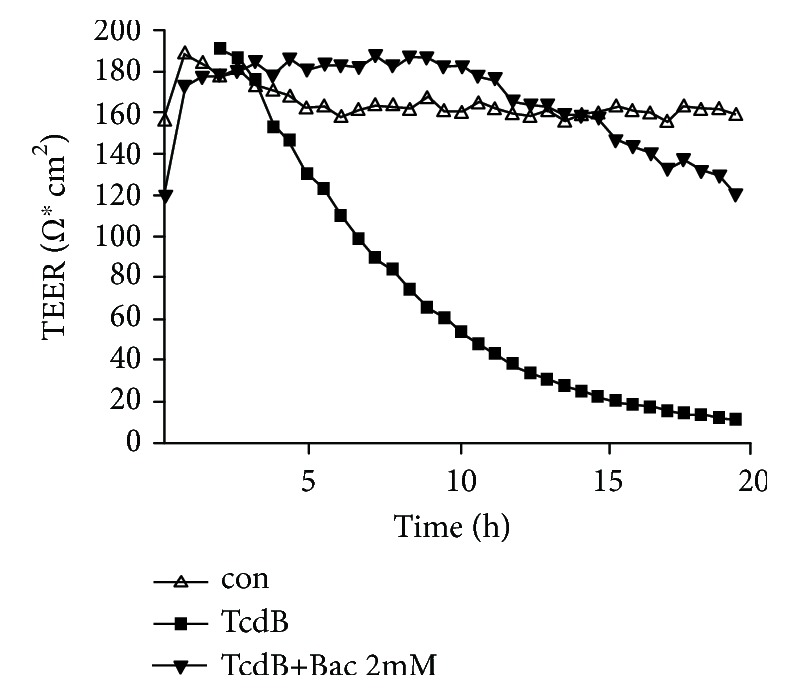
Bac preserves epithelial integrity of CaCo-2 monolayers from TcdB activity. CaCo-2 cells grown as epithelial monolayers on filters in a Transwell chamber were preincubated with or without Bac (2 mM) for 30 min at 37°C. Then, TcdB (6 ng/ml) was added apically. For control, cells were left untreated. The epithelial integrity was recorded by measuring the transepithelial electrical resistance (TEER) every 35 min over a time course of 20 h.

Having demonstrated that Bac protects CaCo-2 cells from TcdB, we next investigated whether Bac also preserves the epithelial integrity of CaCo-2 monolayers in a Transwell chamber, which represents a simplified yet established *in vitro* model for the human gut barrier. By measuring the transepithelial electrical resistance (TEER) over a period of 20 h (Figure 3), it became evident that Bac has a strong protective effect towards TcdB in this model. While treatment of the epithelia with TcdB alone resulted in a rapid decrease of TEER, indicating the loss of barrier function, the TEER values for epithelia treated with TcdB in the presence of Bac were similar to those from untreated cells, even after 20 h of treatment, there was an intact barrier function. This is of particular interest because the toxin-induced loss of barrier function in the human gut is a hallmark in CDAD.

### 3.3. Bac Reduces the TcdB-Induced Glucosylation of Rac1 and Destruction of F-Actin in Human Intestinal Organoids (“Miniguts”)

**Figure 4:**
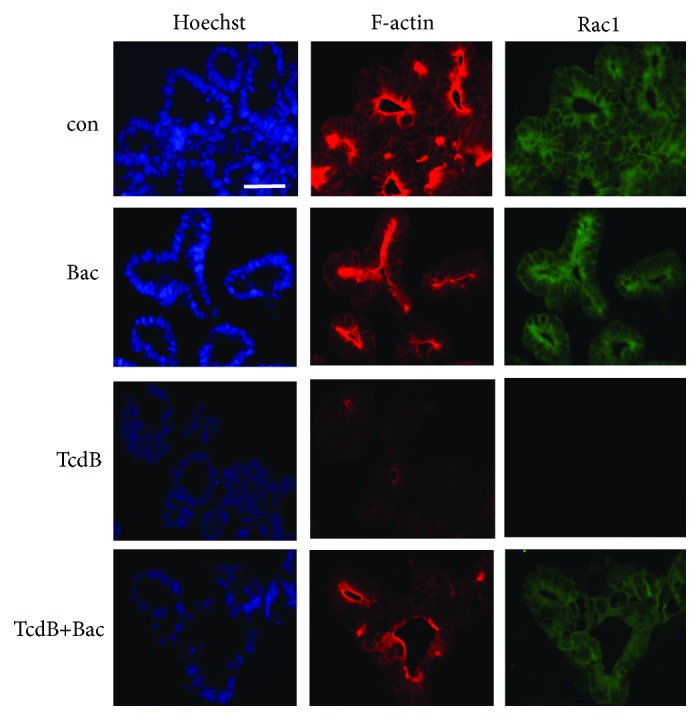
Bac reduces the TcdB-induced glucosylation of Rac1 and destruction of F-actin in human intestinal organoids (“miniguts”). “Miniguts” were preincubated for 30 min at 37°C with or without 1 mM Bac. Then, TcdB (60 ng/ml) was added and the organoids were incubated for further 3 h. For control (con), cells were left untreated. After washing and extraction from Matrigel, “miniguts” were fixed and frozen sections were prepared. Nuclei, F-actin, and nonglucosylated Rac1 were specifically stained as described earlier [2, 3] and visualized by confocal fluorescence microscopy. Bar = 50 *μ*m.

Prompted by these results, we finally investigated the effect of Bac on TcdB in the clinically relevant human “minigut” model. These stem cell-derived intestinal organoids were challenged with TcdB in the absence and presence of TcdB, and the glucosylation status of Rac1 and the F-actin content in the organoids, both endpoints of the cytotoxic mode of action of TcdB, were analyzed by confocal fluorescence microscopy (Figure 4). In organoids treated with TcdB in the absence of Bac, there was no obvious signal for unmodified Rac1 indicating that most of the Rac1 in the cytosol of the cells was glucosylated by TcdB. Moreover, there was only a negligible signal for F-actin in TcdB-treated organoids, which is depolymerized as response to the inactivation of the Rho-GTPases in these cells by TcdB. The loss of F-actin is one major reason for the loss of epithelial integrity and barrier function in the gut. In contrast, the organoids treated with TcdB in the presence of Bac had similar amounts of unmodified Rac1 and F-actin than untreated cells.

## 4. Conclusions

In conclusion, a set of experiments was performed to demonstrate the toxin-neutralizing effects of Bac towards *C. difficile* TcdB in the human intestinal epithelial cells and stem-cell derived human intestinal organoids from this important *C. difficile* toxin, which is a major contributor to CDAD. In combination with the recent observation that Bac neutralizes the actin-modifying toxin CDT from hypervirulent *C. difficile* strains [25], the findings might offer a starting point for the development of novel therapeutic options in the context of CDAD, in particular because Bac is already an approved drug in clinical use [24]. The implementation of iPSC-derived intestinal organoids could further fortify a potential role for Bac in the direct treatment of *C. difficile*-induced toxicity.

## Data Availability

The data (cell and organoid pictures, Western blot X-ray films, and measurements of TEER) are available from the corresponding author upon request.

## Abbreviations

Bac: Bacitracin
Baf: Bafilomycin
*C.*: *Clostridium*
CDAD: *C. difficile*-associated diseases
CDI: *Clostridium difficile* infection
CDT: *C. difficile* transferase (binary toxin)
DMEM: Dulbecco's modified Eagle medium
ECL: Enhanced chemiluminescence
FCS: Fetal calf serum
iPSC: Induced pluripotent stem cell
SD: Standard deviation
SDS-PAGE: Sodium dodecyl sulfate polyacrylamide gel electrophoresis
SEM: Standard error of the mean
TcdA: Toxin A from *C. difficile*
TcdB: Toxin B from *C. difficile*
TEER: Transepithelial electrical resistance.

## References

[1] Perkofer L., Frappart P.-O., Müller M., Kleger A. (2018). Importance of organoids for personalized medicine. *Personalized Medicine*.

[2] Ernst K., Ernst K., Schmid J. (2017). Hsp70 facilitates trans-membrane transport of bacterial ADP-ribosylating toxins into the cytosol of mammalian cells. *Scientific Reports*.

[3] Di Masi A., Leboffe L., Polticelli F. (2018). Human serum albumin is an essential component of the host defense mechanism against *Clostridium difficile* intoxication. *The Journal of Infectious Diseases*.

[4] Bartlett J. G., Gerding D. N. (2008). Clinical recognition and diagnosis of *Clostridium difficile* infection. *Clinical Infectious Diseases*.

[5] Kelly C. P., LaMont J. T. (2008). *Clostridium difficile* — more difficult than ever. *The New England Journal of Medicine*.

[6] Rupnik M., Wilcox M. H., Gerding D. N. (2009). *Clostridium difficile* infection: new developments in epidemiology and pathogenesis. *Nature Reviews. Microbiology*.

[7] Walker A. S., Eyre D. W., Wyllie D. H. (2013). Relationship between bacterial strain type, host biomarkers, and mortality in *Clostridium difficile* infection. *Clinical Infectious Diseases*.

[8] Leffler D. A., Lamont J. T. (2015). *Clostridium difficile* infection. *The New England Journal of Medicine*.

[9] Kuehne S. A., Cartman S. T., Heap J. T., Kelly M. L., Cockayne A., Minton N. P. (2010). The role of toxin A and toxin B in *Clostridium difficile* infection. *Nature*.

[10] Steele J., Chen K., Sun X. (2012). Systemic dissemination of *Clostridium difficile* toxins A and B is associated with severe, fatal disease in animal models. *The Journal of Infectious Diseases*.

[11] Just I., Gerhard R. (2004). Large clostridial cytotoxins. *Reviews of Physiology, Biochemistry and Pharmacology*.

[12] Jank T., Aktories K. (2008). Structure and mode of action of clostridial glucosylating toxins: the ABCD model. *Trends in Microbiology*.

[13] Papatheodorou P., Zamboglou C., Genisyuerek S., Guttenberg G., Aktories K. (2010). Clostridial glucosylating toxins enter cells via clathrin-mediated endocytosis. *PLoS One*.

[14] Pfeifer G., Schirmer J., Leemhuis J. (2003). Cellular uptake of *Clostridium difficile* toxin B. Translocation of the *N*-terminal catalytic domain into the cytosol of eukaryotic cells. *Journal of Biological Chemistry*.

[15] Reineke J., Tenzer S., Rupnik M. (2007). Autocatalytic cleavage of *Clostridium difficile* toxin B. *Nature*.

[16] Papatheodorou P., Barth H., Minton N., Aktories K. (2018). Cellular uptake and mode-of-action of *Clostridium difficile* toxins. *Advances in Experimental Medicine and Biology*.

[17] Just I., Selzer J., Wilm M., von Eichel-Streiber C., Mann M., Aktories K. (1995). Glucosylation of Rho proteins by *Clostridium difficile* toxin B. *Nature*.

[18] Popoff M. R., Rubin E. J., Gill D. M., Boquet P. (1988). Actin-specific ADP-ribosyltransferase produced by a *Clostridium difficile* strain. *Infection and Immunity*.

[19] Gerding D. N., Johnson S., Rupnik M., Aktories K. (2014). *Clostridium difficile* binary toxin CDT: mechanism, epidemiology, and potential clinical importance. *Gut Microbes*.

[20] Dickerhof N., Kleffmann T., Jack R., McCormick S. (2011). Bacitracin inhibits the reductive activity of protein disulfide isomerase by disulfide bond formation with free cysteines in the substrate-binding domain. *The FEBS Journal*.

[21] Karala A. R., Ruddock L. W. (2010). Bacitracin is not a specific inhibitor of protein disulfide isomerase. *The FEBS Journal*.

[22] Ming L. J., Epperson J. D. (2002). Metal binding and structure-activity relationship of the metalloantibiotic peptide bacitracin. *Journal of Inorganic Biochemistry*.

[23] Ikai Y., Oka H., Hayakawa J. (1995). Total structures and antimicrobial activity of bacitracin minor components. *The Journal of Antibiotics*.

[24] Dudley M. N., McLaughlin J. C., Carrington G., Frick J., Nightinglale C. H., Quintiliani R. (1986). Oral bacitracin vs vancomycin therapy for Clostridium difficile—induced diarrhea. A randomized double-blind trial. *Archives of Internal Medicine*.

[25] Schnell L., Felix I., Müller B. (2019). Revisiting an old antibiotic: bacitracin neutralizes binary bacterial toxins and protects cells from intoxication. *The FASEB Journal*.

[26] Huelsenbeck S. C., Klose I., Reichenbach M., Huelsenbeck J., Genth H. (2009). Distinct kinetics of (H/K/N)Ras glucosylation and Rac1 glucosylation catalysed by *Clostridium sordellii* lethal toxin. *FEBS Letters*.

[27] Hohwieler M., Renz S., Liebau S. (2016). “Miniguts” from plucked human hair meet Crohn’s disease. *Zeitschrift für Gastroenterologie*.

[28] Hohwieler M., Illing A., Hermann P. C. (2017). Human pluripotent stem cell-derived acinar/ductal organoids generate human pancreas upon orthotopic transplantation and allow disease modelling. *Gut*.

